# Successful Management of a Rare Manifestation of Intramuscular Venous Malformation in a Young Adult: A Case Report

**DOI:** 10.7759/cureus.37812

**Published:** 2023-04-19

**Authors:** Chinmayi S Sharma, Sanath N Bhandari, Mayur Rai

**Affiliations:** 1 Department of Orthopaedics, AJ Institute of Medical Sciences and Research Centre, Mangalore, IND; 2 Department of Plastic and Reconstructive Surgery, AJ Institute of Medical Sciences and Research Centre, Mangalore, IND

**Keywords:** congenital vascular lesion, flexion deformity, delayed diagnosis, knee deformity, intramuscular venous malformation

## Abstract

Venous malformations are the most common type of congenital vascular lesions resulting from abnormal embryonic development of vessels. Typical venous malformations are easily diagnosed by skin color changes, focal edema, or pain as they are mostly present in the skin and subcutaneous tissue. Venous malformations in the skeletal muscles, however, have the potential to be missed because their involved sites are invisible. We describe a 15-year-old patient with extensive intramuscular venous malformations in the lower extremity with special emphasis on diagnosis and treatment.

## Introduction

Vascular anomalies in children are of two varieties: proliferative or vascular tumors (eg. hemangiomas) and vascular malformations [1]. Vascular malformations are congenital heterogeneous conditions characterized by abnormal vessel development [2]. They can be broadly classified into slow-flow (capillary, venous and lymphatic) and fast-flow (arteriovenous) malformations. Combined malformations (venolymphatic) are characterized by multiple vessel involvement. A congenital mixed vascular malformation syndrome known as Klippel-Trenaunay syndrome presents with capillary malformations, venous/lymphatic anomalies and bony/soft tissue hypertrophy [3]. Venous malformations (VM) are the most common type of vascular malformation affecting about 1-4% of individuals [4]. VMs in skin or subcutaneous tissue typically diagnosed at birth are benign and often regress during childhood. However, these malformations can be missed when present in skeletal muscles. Affected patients may present later in life with complaints of pain, loss of function, infection, coagulopathy or even psychological challenges [1]. A thorough assessment of such patients with a high index of suspicion followed by magnetic resonance imaging (MRI) and angiography are useful tools for diagnosing these lesions.

Herewith, we report a case of a deep intramuscular VM (IMVM) that went undiagnosed in a paediatric patient until he was older but was successfully managed with surgical intervention and rehabilitation.

## Case presentation

**Figure 1:**
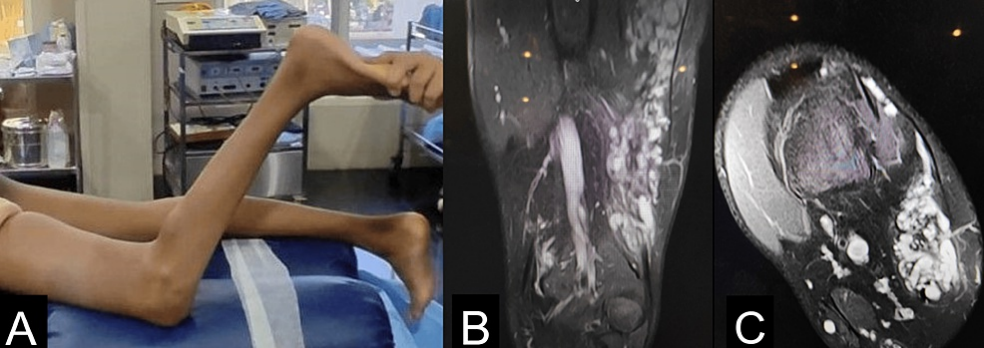
Intramuscular venous malformation (IMVM) involving the left hamstrings in our patient demonstrating (A) pre-operative severe fixed flexion deformity, Magnetic resonance images of involved leg showing (B) multiple engorged tortuous venous structures involving the quadriceps muscles with abnormal venous contrast enhancement (C)

A 15-year-old male was evaluated for left knee pain and difficulty walking for the past six years. He did not have any history of previous trauma or other pertinent past medical or surgical illnesses. Physical examination demonstrated, a severe flexion deformity, with a range of motion of −90° - 135° of flexion in the left knee (Figure 1A). Wasting of muscles noted over left calf and thigh. The left patella was fixed, but otherwise symmetrical in both limbs. He had a normal range of motion in his right knee and bilateral ankle and hips. Investigative workup in the form of an X-ray revealed reduced left knee joint space. MRI of the leg showed multiple engorged tortuous venous structures, primarily involving the hamstrings (biceps femoris, semitendinosus, and semimembranosus) and vastus lateralis (Figure 1B, 1C). CT cuts revealed atrophy of hamstring muscles and vastus lateralis with punctuate calcifications.

After discussing treatment options with the family, intervention radiology, cardio-thoracic and vascular surgery teams, surgical resection of the venous malformations was performed. The surgery was performed under general anesthesia in prone position on a regular operating table. The incision was placed on the posterior aspect of the thigh, from mid-thigh extending distally. After blunt dissection of the layers, care was taken to identify and preserve the sciatic nerve. Sparing the tendons, necrosed parts of hamstring muscles and vastus lateralis were identified and excised along with the supplying vessels. 

**Figure 2:**
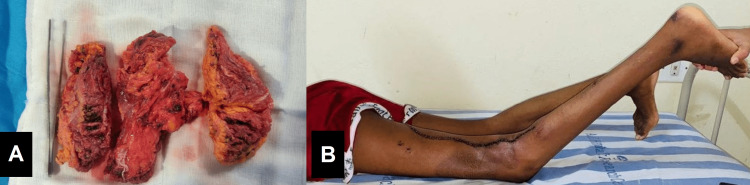
(A) Resected specimen showing skeletal muscles, adipose tissue and inflammatory changes and (B) postoperative improvement of deformity prior to cast application.

Resected specimen (of fibromuscular soft tissue with attached subcutaneous adipose tissue measuring 20x12x6cm) showed skeletal muscle fibers, adipose tissue, numerous irregularly elongated, dilated, and thick-walled capillaries with chronic inflammation suggestive of arterio-venous malformation on histopathology (Figure 2A). This was followed by an above-knee cast application in maximum extension as possible, considering the neurovascular stretch injury (Figure 2B). The patient underwent four such serial castings under general anesthesia over three months.

**Figure 3:**
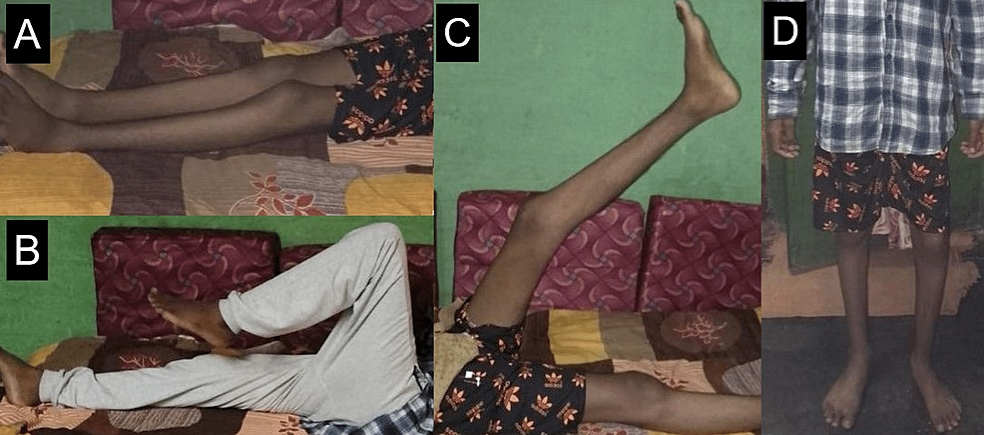
Post-rehabilitation pictures (A) showing complete extension in the operated limb, (B) and (C) showing a full range of motion and (D) stance without assistance.

At the end of three months, our patient was able to achieve complete extension (Figure 3A). His cast was removed and he was initiated on full weight-bearing mobilization. He underwent rehabilitation for three months and is now ambulant without assistance. On follow-up examination, no deformities were noted. The patient has a range of motion of 0° - 135° of flexion in his left knee (Figure 3B, 3C). He does not complain of pain or discomfort on walking (Figure 3D).

## Discussion

Intramuscular venous malformations can result in severe co-morbidities if left undiagnosed for a long time. The diagnosis of this condition, however, is very challenging due to their young age at presentation and due to the deep location of the lesions [5]. Hence, a high index of suspicion followed by appropriate imaging is warranted to help with prompt diagnosis and management. This is particularly challenging in resource-limited settings where the diagnosis may be delayed due to the lack of knowledge regarding this condition and the lack of appropriate resources. Despite the delay in diagnosis, our patient was able to make a full recovery when presented with the necessary expertise from the surgical and radiology team.

Though intramuscular VMs may be present at birth and are believed to be benign, they have the potential to grow in children during periods of their growth [6]. They are frequently asymptomatic with pain at the site of the lesion often being the presenting symptom. Differential diagnosis of the pain includes traumatic injury, myositis, or fasciitis. Delayed diagnosis of this condition is due to its involved sites being invisible and its presentation with obscure symptoms. An accurate diagnosis of the VMs though can be made with a Doppler ultrasound coupled with an MRI of the affected extremity [7]. Treatment of the condition includes observation and active surveillance of asymptomatic or mildly symptomatic lesions, sclerotherapy, or surgical resection of the VMs [4]. Sclerotherapy is a non-surgical intervention that may be considered for smaller lesions [8]. However, this may be associated with recurrence, local fibrosis, and development of contractures. Surgical resection followed by rehabilitation has been shown to have the best outcomes in a recent study by Kim and colleagues. In this study, the benefit from surgery was particularly noted with respect to improvement in extremity movements and radiological response. Soft tissue and skeletal overgrowth is a common problem in case of vascular malformation with lesions especially when present around growth plates/joints of the long bones. Overgrowth in the lower limb can cause a limp, pain and may develop into a flexion deformity if left untreated [4]. Surgical excision with deformity correction is the most effective option, whenever the clinical entity is recognized unless contraindicated. Though surgical resection gives very good results, surveillance is recommended to monitor for recurrence of the lesion.

## Conclusions

IMVMs can be difficult to diagnose and treat, due to delayed presentation, deep location and vague symptoms. A thorough physical examination, followed by an appropriate investigative workup is necessary for timely diagnosis. Most IMVMs are asymptomatic and some may present with pain at the site of the lesion. In that case, traumatic injury, myositis and fasciitis must be ruled out. It is also crucial to be able to differentiate a vascular malformation from a malignancy. In a lesion that presents as a mass in an extremity, an MRI followed by a biopsy of the lesion should be done if the diagnosis is suspicious. On diagnosis of an IMVM, a treatment plan is to be formulated, keeping in mind the patient's age, presenting symptoms, location of the lesion, and extent of the deformity. In our patient, a surgical excision with deformity correction followed by regular follow-up resulted in full recovery. Therefore, though our patient’s condition went undiagnosed for a long time due to the lack of awareness and his limited resources, when presented with opportunities to avail the necessary expertise, a prompt diagnosis and successful intervention were done to help with a complete recovery from his presentation of a large intramuscular venous malformation.
